# Signaling via C-C Chemokine Ligand 19 and Extracellular Regulated Kinase 5 in T Cells Limits the Humoral Adaptive Immune Response in Mice

**DOI:** 10.3390/ijms26199744

**Published:** 2025-10-07

**Authors:** Jaisel A. Cervantes, T. Paul Welch, Brian Kaiser, Charles A. Bill, Angel Torres, Gareth L. Bill, Colin A. Bill, Charlotte M. Vines

**Affiliations:** 1 Department of Biological Sciences, The University of Texas at El Paso, El Paso, TX 79968, USA; jacervantes5@utep.edu (J.A.C.); atorres118@utep.edu (A.T.); cabill@utep.edu (C.A.B.); 2 Department of Microbiology, Molecular Genetics and Immunology, The University of Kansas Medical Center, Kansas City, KS 66103, USA; medhelix@gmail.com (T.P.W.); briandkaiser@gmail.com (B.K.)

**Keywords:** extracellular regulated kinase 5, C-C chemokine receptor 7, autoimmunity, C-C chemokine ligand 19

## Abstract

Misregulation of C-C chemokine receptor 7 (CCR7) has been linked to multiple autoimmune diseases including systemic lupus erythematosus, multiple sclerosis, and ankylosing spondylitis. As a G-protein-coupled receptor, located on the cell membrane, CCR7 can be targeted by inhibiting one of its two ligands, C-C chemokine ligand 19 (CCL19), to regulate its function. In this study, we examined signaling events downstream of CCL19 binding that provide a mechanism for regulation of the immune response. We used a CCR7 antagonist, CCL19_8-83_, in immune studies in vivo, as a platform for a pharmaceutical to define the molecular events that are involved in regulating the humoral adaptive immune response. We found that in the presence of a T-cell-dependent antigen, C57BL/6 mice treated during antigen exposure with CCL19_8-83_ generated significantly higher levels of IgG1, the dominant isotype in extracellular bacterial infections that can activate complement, and IgG2c, the dominant isotype during viral and intracellular bacterial infections. Inhibiting ERK5 signaling downstream of CCR7 activation by CCL19, or disruption of CCL19 expression in CCL19^−/−^ mice, also resulted in higher levels of IgG1 when compared to control mice. Differences in levels of IL-4 or other cytokines or lymphocyte types between wild-type and ERK5-deficient T cells did not account for antibody levels. Since pertussis-toxin-induced inhibition of lymphocyte chemotaxis is linked to elevated levels of IgG, we examined the effect of ERK5 on chemotaxis to CCR7 ligand CCL19. We found that disruption of ERK5 in T cells, or global disruption of CCL19 or CCR7, inhibited chemotaxis of T cells to CCL19, a mechanism that enhances sensitization during the exposure to an immunogen. Since CCR7 and its ligands have been linked to autoimmunity, these studies may provide insight into mechanisms that can be targeted to control autoimmune responses.

## 1. Introduction

One out of every seven Americans lives with an autoimmune disease, such as systemic lupus erythematosus, rheumatoid arthritis, or polymyositis, diseases in which autoantibodies against the target tissues are produced during the disease inception. These diseases are linked to misregulation of C-C chemokine receptor 7 (CCR7) and a dysregulated adaptive immune response [1]. The mechanisms that promote these autoimmune disorders through misregulation of CCR7 are not well understood. CCR7 is expressed in naïve and central memory T cells, naïve B cells, monocytes, activated antigen-presenting cells, and neutrophils, where it promotes migration of these immune populations to the lymph nodes and other secondary lymphoid tissues [2,3,4].

In humans, CCR7 responds to three endogenous ligands, C-C chemokine ligand 19 (CCL19), CCL21, and a naturally occurring CCL21 derivative (C21^TP^) that is generated at sites of inflammation [5]. Under homeostatic conditions, CCL21 is expressed on high endothelial venules where it promotes entry of T cells into the lymph nodes [6,7], while during inflammation, CCL19 is expressed on activated dendritic cells (DC), activated B cells, or within the lymph nodes [8], where it can promote the recruitment of naïve T cells [9,10]. Within lymph nodes, T cells survey activated DCs for peptides within their major histocompatibility (MHC) complexes that bind to the T-cell receptors (TCRs) of the naïve T cells. Once a T cell recognizes a peptide/MHC complex, the T cell matures to an effector cell that coordinates antibody isotype switching and affinity maturation [11,12,13]. Although activated dendritic cells and lymph node endothelial cells express CCL19, the role of this ligand in regulating the adaptive immune response has not been well defined.

In mice, CCR7 has three ligands, CCL19, CCL21-leu, and CCL21-ser. While in mice, CCL21-ser attaches to the luminal surfaces of thymic epithelial cells and high endothelial venules (HEVs) of lymph nodes, CCL21-leu is primarily expressed in the lymphatic endothelia of peripheral tissues [14]. In humans, CCL19 is generated in the cytoplasm of high endothelial vessels (HEVs), where it is transported to the apical surface to facilitate T-cell recruitment and arrest [15]. During the adaptive immune response, CCL19 promotes adhesion of T cells to activated antigen-presenting cells, such as dendritic cells, which express this ligand on their surfaces to initiate the adaptive immune response. Concomitantly, activated B cells migrate to the edge of the B-cell follicle, promoting T-cell interactions. Although CCR7 has been shown to limit levels of IgG2a and IgG2b during the adaptive immune response in BALB/c mice [4], it remains unclear to what extent CCL19 binding to CCR7 on naïve T cells contributes to this response. Therefore, in this study, we examined the role of CCL19 and its downstream signaling in regulating the adaptive immune response.

We have shown that in T cells, downstream from CCR7 binding to CCL19 and to a lesser extent CCL21, the concentration of extracellular regulated kinase 5 (ERK5, also known as mitogen-activated protein kinase 5 (MAPK5)) in the cells increases, along with the phosphorylation state of this MAPK. ERK5 phosphorylation is sustained for 72 h following T-cell exposure to CCL19, during which time this transcription factor is translocated to the nucleus [8]. The role of ERK5 signaling in regulating the immune response is controversial. While we have shown that downstream from IL-17 pro-inflammatory activation, ERK5 stimulates cellular proliferation [16], a pharmacological inhibitor study demonstrated that drug-induced degradation of ERK5 had no effect on secretion of the pro-inflammatory cytokine interleukin-8 (Il-8) by endothelial cells, nor did the degradation of ERK5 affect cellular proliferation [17]. In contrast, ERK5 expression in macrophages promoted atherosclerosis and exacerbated inflammation via neutrophil netosis [18,19]. While ERK5 is activated downstream of CCR7/CCL19, the role of expression and activation of ERK5 downstream of CCR7 during inflammation is unclear.

In this study, we examine the role of CCR7 signaling via CCL19 during the adaptive immune response to the T-cell-dependent, 2,4-dinitrophenyl (DNP) hapten conjugated to keyhole limpet hemocyanin (DNP-KLH) to evaluate the role of CCL19 and signaling downstream to ERK5 in regulating the secondary immune response. We find that while CCR7 limits production of all four IgG isotypes (IgG1, IgG2b, IgG2c, IgG3) in C57BL/6 mice during the adaptive immune response, CCL19 only limits the levels of IgG1 and IgG2c during the primary and IgG1 alone during secondary immune responses. Downstream of CCL19 binding to CCR7, we found that ERK5 promotes migration of cells to CCL19. Using ERK5 conditional knockout mice, we found that ERK5 expression in T cells limits levels of IgG1 and IgG2c when compared to levels produced by control mice during the adaptive immune response to DNP-KLH. Specifically, we found that loss of CCR7, its ligand CCL19, or its downstream effector ERK5 disrupted chemotactic migration of lymphocytes to CCL19, a process associated with elevated titers of IgG1 [20]. This is the first report of a role linking CCL19/CCR7 signaling through ERK5 to induce chemotaxis to regulating the levels of IgG1 and IgG2c produced during the humoral adaptive immune response.

## 2. Results

### 2.1. CCR7 Signals to Limit the Extent of the Secondary Immune Response

**Figure 1:**
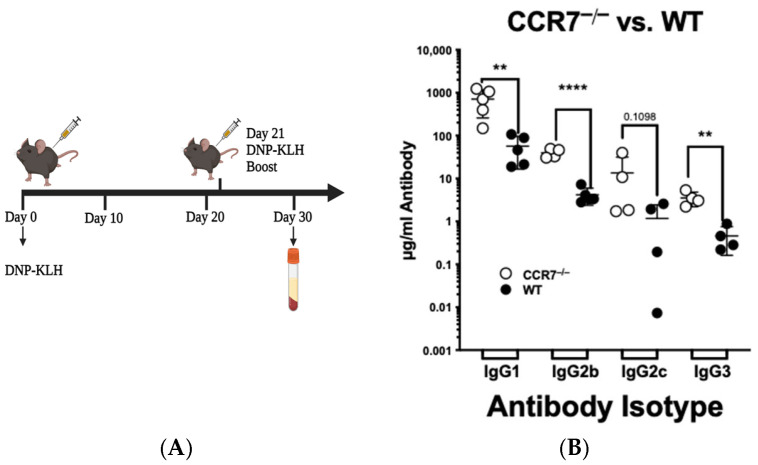
CCR7 limits the extent of the adaptive immune response in C57BL/6 mice. (**A**) Injection scheme for 200 µg of DNP-KLH. (**B**) ELISA data of anti-DNP antibodies generated on day 30. Horizontal lines indicate arithmetic means for each group of animals. Each symbol represents a mouse (4–5 mice were used per group). Representative of *n* ≥ 3 independent experiments. Significance was determined for each mouse using paired Student’s *t*-test (** *p* < 0.01, **** *p* < 0.0001).

CCR7^−/−^ mice have an enhanced response to the T-dependent antigen DNP-KLH. CCR7^−/−^ BALB/c mice, which skew towards a T helper cell 2 (Th2) response, produced an enhanced humoral adaptive immune response, with higher levels of anti-DNP IgG2a and IgG2b in the CCR7^−/−^ BALB/c mice after the booster, when compared to wild-type controls. To establish that CCR7^−/−^ mice on the Th1-skewed C57BL/6 background produce an enhanced humoral adaptive immune response, wild-type and CCR7^−/−^ mice were immunized i.p. with DNP-KLH and boosted on day 21 (Figure 1). Along with IgG3, IgG1, the dominant isotype in extracellular bacterial infections that can activate complement, and IgG2c, the dominant isotype during viral and intracellular bacterial infections are elevated [21]. We found that 10 days after the booster, the levels of IgG1, IgG2b, and IgG3 were significantly higher in the CCR7^−/−^ mice than in WT mice, and levels of IgG2c were trending towards significance as well. These data demonstrate that in the Th1-skewed background of the C57BL/6 mouse, CCR7 limits the levels of antibodies produced in response to a T-dependent antigen.

### 2.2. CCL19 Limits Levels of IgG1

**Figure 2:**
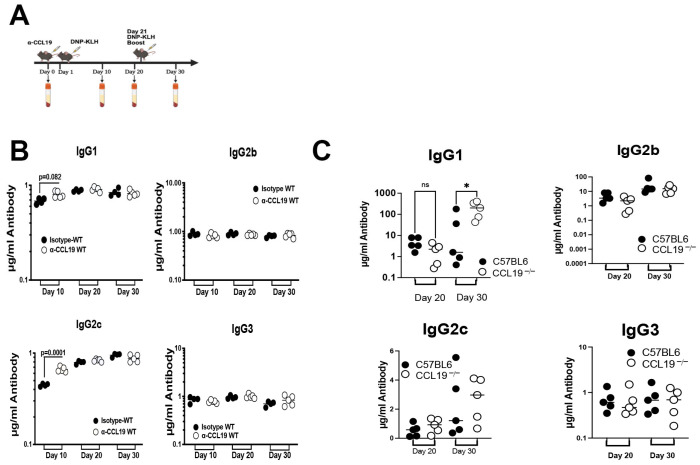
CCL19 limits the extent of the adaptive immune response. (**A**) Injection scheme: (**B**) Each C57BL/6 mouse was injected i.p. with 100 µg of anti-CCL19 or isotype control antibody and allowed to rest for an hour. (**B**) C57 BL/6 wild-type mice or (**C**) C57 BL/6 mice homozygously deleted for CCL19 (CCL19^−/−^) were injected at the base of the tail on day 1 and boosted on day 21 with 200 µg of DNP-KLH. Submandibular bleeds for ELISA measurements were collected on days 10, 20, and 30; sera were transferred to a fresh tube following centrifugation and used immediately or stored at −80 °C for future analysis. Sandwich ELISA for anti-DNP antibodies was used to measure levels of IgG1, IgG2b, IgG2c, or IgG3 on days 10, 20, and 30 as described in the methods. Each symbol represents antibody levels in a mouse. Horizontal lines indicate arithmetic means for each group of animals (5 mice were used per group). Significance in the changes between wild-type and test mice was determined for each pair of IgG readings using paired Student’s *t*-test comparing absence of CCL19 to control (* *p* < 0.05, ns = values that are not significant).

T cells, recruited to the lymph nodes via CCR7 during homeostasis and during an immune response [22,23,24], play central roles in regulating the levels of antibody isotypes produced during an adaptive immune response [4,25,26,27]. CCL19 promotes adhesion of primary T cells to dendritic cells [8], yet it is unclear if CCL19 signaling has an additional role during the humoral immune response. To examine the role of CCL19 in the adaptive immune response, we injected mice with anti-CCL19 function-blocking/neutralizing antibodies [28]. Since these function-blocking antibodies originated in a goat, we used only a single injection to block CCL19 to avoid the possibility of serum sickness. During the primary immune response, we observed higher levels of IgG1 and IgG2c when compared to wild-type mice; there was no elevation in IgG2b or IgG3 (Figure 2B). We found that there was no change in secondary immune response between anti-CCL19 and the wild type, but this is likely due to loss of anti-CCL19 effects. We also compared the primary and secondary immune responses in CCL19^−/−^ versus control C57BL/6 mice and found that there was no difference in the primary response; however, we did observe a significant increase in levels of IgG1 during the secondary immune response (Figure 2C).

**Figure 3:**
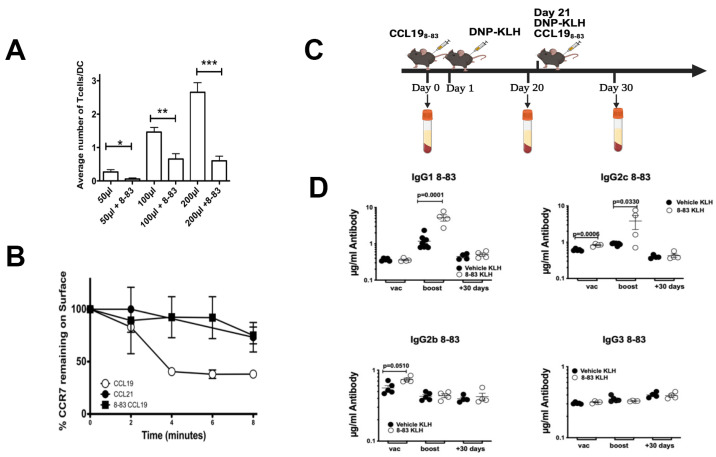
CCL19 antagonist, CCL19_8-83_, inhibits CCR7/CCL19-mediated functions of T cells and enhances the production of IgG1. (**A**) TNF-α-matured, CCL19-expressing mature BMDDCs (mBMDDCs) were loaded with ovalbumin and labeled with CMTMR. The mBMDDCs were co-incubated with 5 × 10^4^, 10^5^, or 2 × 10^5^ of CSFE-stained PBMCs ± CCL19_8-83_ at 37 °C for four hours, at which point adherent cells were counted. Columns indicate the number of PBMCs bound to each mBDDC. (**B**) T cells were induced to internalize CCR7 in the presence of 200 nM CCL19 ± 1µM CCL19_8-83_ for 2, 4, 6 or 8 min, plunged into ice-cold PBS, stained with FITC-anti-CCR7, and analyzed for CCR7 levels remaining on the surface. (**C**) Injection scheme. (**D**) ELISA data generated from mice treated with CCL19_8-83_ on days 0 and 21, followed by immunization with 200 µg of DNP-KLH on days 1 and 21. Blood was collected and analyzed for the total levels of anti-DNP antibodies present in the serum by ELISA on days 20, 50, and 80. Horizontal lines indicate arithmetic means for each group of animals; each point represents an animal (4–6 mice were used per group); *n* = 2 independent experiments. For figures (**A**,**D**), paired Student’s *t*-tests were used to determine significance of differences between groups ((**A**) * *p* < 0.05, ** *p* < 0.01, *** *p* < 0.001).

To avoid immune response against allogenic goat anti-mouse CCL19 antibodies, we used a CCL19 antagonist, CCL19_8-83_, which selectively antagonizes CCL19 but not CCL21 [29,30], to inhibit CCR7/CCL19 association. Previous studies have shown that CCL19 promotes conjugation of T cells with activated DCs [8], which is an important step in priming T cells within lymph nodes. For these studies, we used OTI-T cells, which are CD8+T transgenic T cells that are designed to recognize ovalbumin, when it is presented by antigen-presenting cells on the surface by MHC class I [8,31]. To confirm that synthetic CCL19_8-83_ could block adhesion of DCs to T cells, we measured the binding of DCs to increasing concentrations of OTI T cells in the presence of a two-fold excess of the IC50 (1.1 µM) CCL19_8-83_. An equal volume of vehicle (PBS/5% BSA) was used as the control. Since activated DCs express CCL19 on the surface, we hypothesized that in the presence of CCL19_8-83_, we would be able to reduce the conjugation of T cells with their cognate BMDDCs. Ovalbumin-primed BMDDCs were co-incubated with OTI T cells and allowed to form conjugates in the presence of increasing numbers of cells ± CCL19_8-83_ (Figure 3). We found that there were significantly fewer T cells bound to BMDDCs in the presence of CCL19_8-83_ than the control in the presence of vehicle (Figure 3A). To confirm that CCL19_8-83_ blocked CCL19 function, we measured internalization of CCR7/CCL19 and CCR7/CCL21 in the presence of CCL19_8-83_ in T cells. While CCL19_8-83_ blocked internalization of CCL19, previous studies have shown that CCL19_8-83_ has no effect on internalization of CCL21 [29,30].

Subsequently, we used the CCL19_8-83_ peptide to determine to what extent CCL19 contributed to the enhanced production of IgGs found in CCR7^−/−^ mice, when compared to wild-type control, following injection with a T-dependent immunogen DNP-KLH. Study mice were injected with 100 µg of CCL19_8-83_ on day 0, while control mice were injected with vehicle/DNP-KLH. After 24 h, all mice were immunized with 100 µg of DNP-KLH. Mice that received CCL19_8-83_ on day 0 were then boosted with CCL19_8-83_/DNP-KLH, while control mice were treated with vehicle/DNP-KLH on day 21. To determine at what point antibody levels returned to normal, we extended our assay and measured production of DNP-specific antibodies at 20, 50, and 80 days following initial immunization. We observed that only IgG2c was significantly increased when compared to levels found in wild-type mice within 20 days, and that both IgG1 and IgG2c were significantly increased when compared to levels found in wild-type mice within 50 days (Figure 3D). All antibody isotype levels returned to the levels found in wild-type mice by day 80.

### 2.3. CCR7 Regulation of ERK5 Controls Antibody Production

**Figure 4:**
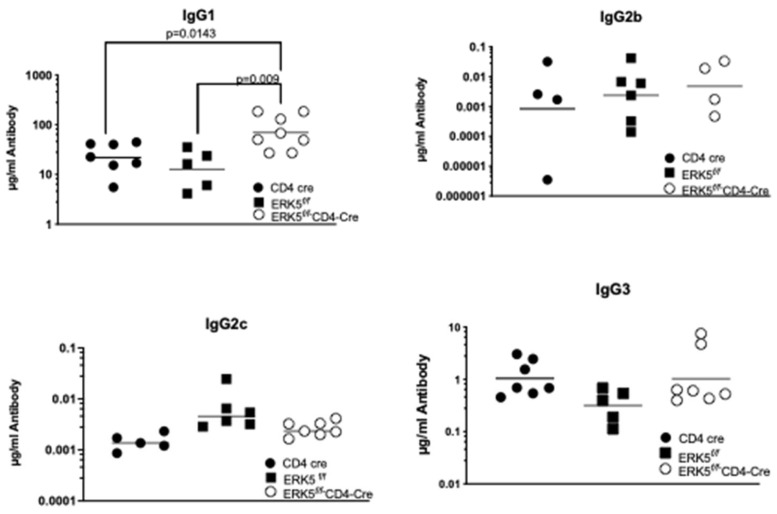
ERK5*^f/f^*CD4-Cre mice enhance production of IgG1 subclass. ERK5*^f/f^* mice and ERK5*^f/f^*CD4-Cre mice were immunized s.c. at the base of the tail with 200 µg of DNP-KLH, and sera were collected on day 30 (see schematic and description of experiment in Figure 2). Each symbol represents antibody levels in a mouse. Horizontal lines indicate arithmetic means for each group of animals (4–7 mice were used per group). Significance between wild-type control mice (ERK5*^f/f^* or CD4-Cre) and test mice (ERK5*^f/f^*CD4-Cre) was determined for each pair of IgG levels using paired Student’s *t*-test. *n* = 3 independent experiments. Significance is *p* < 0.05.

We have shown that CCL19/CCR7 promotes signaling to increase expression, phosphorylation, and subsequent nuclear translocation of ERK5 in T cells [8]. To determine if this CCL19/CCR7-induced ERK5 signaling in T cells limits levels of IgG1, similar to the CCL19^−/−^ mice, we used ERK5 conditional knockouts in T cells. For these studies, ERK5*^flox/flox^* (ERK5*^f/f^*) mice were crossed to the CD4-Cre mice, where all thymocytes mature through a double-positive stage and express CD4 and CD8 before becoming single-positive. We compared antibody production in the ERK5*^f/f^* mice to that of the ERK5*^f/f^*CD4-Cre (Figure 4). We found no significant differences in the levels of IgG antibodies generated 10 days after the primary immunization. However, 10 days following the boost, we found that the ERK5*^f/f^*CD4-Cre mice generated significant increases in the levels of IgG1, when compared to ERK5*^f/f^* controls. Like what was observed in mice treated with CCL19_8-83_, there were also enhanced levels of IgG2c 10 days after immunization; however, these levels were not sustained and resolved over time. We concluded that CCL19/CCR7 signaling through ERK5 in T cells limits levels of IgG1 and IgG2c during the secondary adaptive humoral immune response.

### 2.4. Cytokines Regulated by ERK5 Do Not Limit the Immune Response

**Figure 5:**
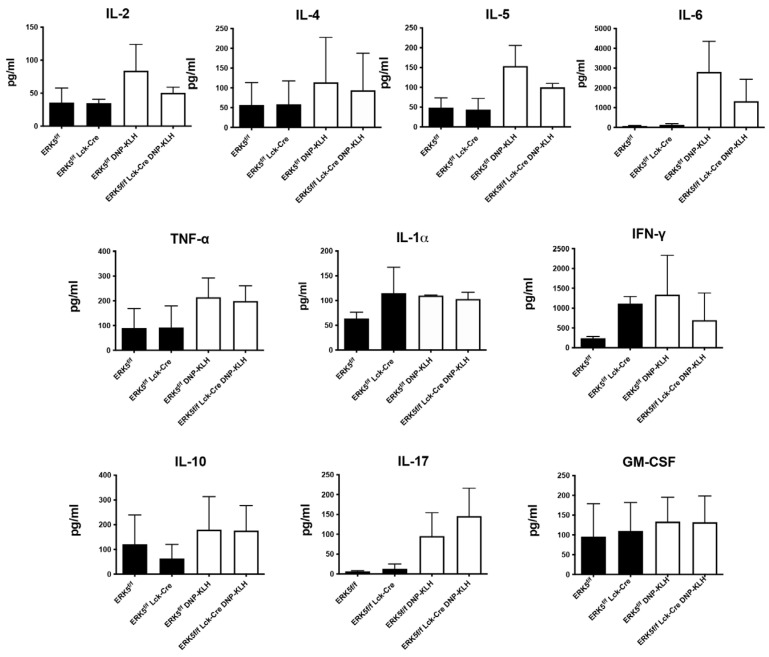
Chemokine production does not vary significantly between ERK5*^f/f^* Lck-Cre and ERK5*^f/f^* T cells. Splenocytes were isolated from ERK5*^f/f^*CD4-Cre and ERK5*^f/f^* mice and allowed to proliferate in the presence of IL-2 and IL-7 with (white) or without (black) DNP-KLH for 24 h in vitro. Levels of cytokines expressed in the supernatants were measured using a flow-cytometry-based bead assay. Columns represent the mean expression levels of individual cytokines ± SD for 2 or more independent experiments, with measurements made from ≥6 mice. Paired Student’s *t*-tests were used to determine significance in differences of levels of cytokines produced by control (ERK5*^f/f^*) versus ERK5-negative (ERK5*^f/f^*CD4-Cre) T cells.

During antibody production, certain interleukins such as interleukin-2 (IL-2) promote T-cell proliferation, and IL-4 and IL-5 contribute to maturation of B cells. To determine if differences in levels of cytokines produced downstream of ERK5 regulated the immune response, we used splenocytes isolated from ERK5*^f/f^*Lck-Cre vs. ERK5*^f/f^* control mice for in vitro recall assays. For ERK5*^f/f^*Lck-Cre, when compared to ERK5*^f/f^* control mice, there were no significant differences in levels of IL-2, IL-4, or IL-5 that could account for enhanced production of IgG1 or IgG3 in the absence of ERK5 (Figure 5) when compared to controls. Similarly, IL-6 promotes T-cell help during antibody production and enhances production of all subclasses of human IgGs [32], yet increases in levels of IL-6 in the absence of ERK5 when compared to control mice did not account for differences in levels of IgGs. We questioned whether pro-inflammatory cytokines tumor necrosis factor a (TNFα), interferon γ (IFNγ), IL-1α, and IL-17 induced an increase in the inflammatory state in the presence of splenocytes that lack ERK5 when compared to the controls. We found no significant differences in the production of these cytokines ± ERK5. In addition, since changes in levels of anti-inflammatory IL-10 could result in differences in levels of antibodies produced, we measured IL-10, which revealed no significant difference in levels of expression independent of ERK5 expression in mice. Cytokines, such as GM-CSF, that stimulate proliferation of myeloid cells that could increase levels of antigen-presenting cells were not modulated independent of ERK5 expression. Overall, we found that there were no significant increases or decreases in cytokine levels that were dependent on ERK5 expression that could account for the selective increase in IgG1 and IgG2c during the adaptive immune response.

### 2.5. B-Cell Populations Do Not Differ in Response to Signaling via ERK5

**Figure 6:**
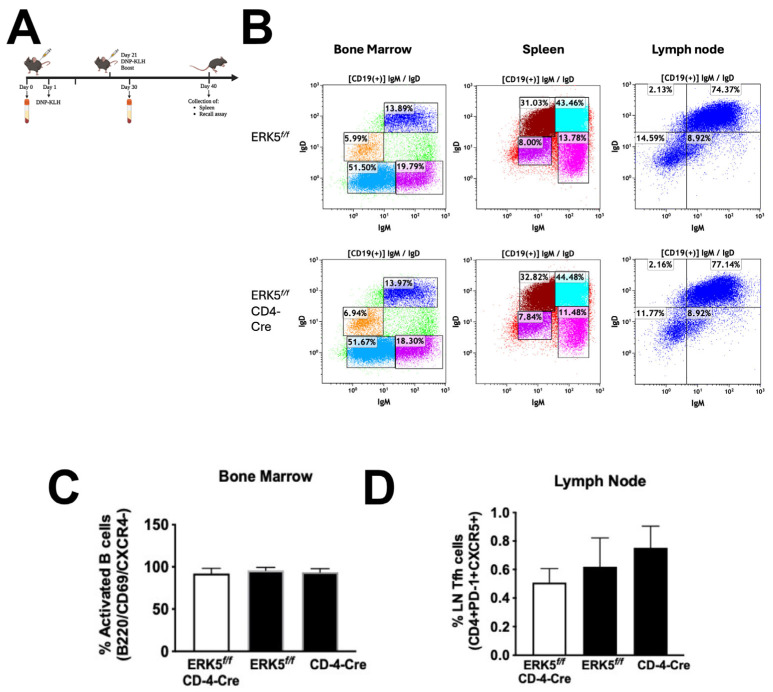
B- and T-lymphocyte populations do not vary significantly between ERK5*^f/f^*CD4-Cre and ERK5*^f/f^* mice. (**A**) Mice were immunized by injection of 200 µg of DNP-KLH at the base of the tail (100 µL/side of the base), on days 0 and 21. Cells were isolated from lymphoid tissues on day 30, from the immunized/boosted ERK5*^f/f^* CD4-Cre and ERK5*^f/f^* mice, and restimulated in vitro in the presence of 100 µg/mL DNP-KLH for 24 h. (**B**) Bone marrows, splenocytes, and lymph nodes were collected and stained for CD19, B220, IgM, and IgD and analyzed by flow cytometry. B220+ cells were gated for CD19+ cells, and levels of IgM^hi^IgD^lo^ (activated) B cells were quantified by flow cytometry. (**C**) Bone marrow cells were stained for CD19, B220, CD69, and CXCR4. B220+ cells were gated for CD69+ cells. CD69+CXCR4− cells were quantified. (**D**) To assay for lymph node follicular helper T cells (Tfh), cells were stained for CD3, CD4, PD1, and CXCR5. CD3+/CD4+-gated T cells were assayed for PD-1/CXCR5 levels. Data is representative of the mean ± SD for 4–6 mice per condition. Differences in the (**C**) levels of activated B cells or (**D**) follicular helper T cells (T_fh_) found in mice with ERK5(−) T cells (white) or ERK5(+) T cells (black) were compared using paired Student’s *t*-test.

Since ERK5 expression in T cells can limit production of IgG1 and IgG3 during the secondary adaptive immune response (Figure 4), we questioned whether changes in the state of the memory B-cell population, influenced by T cells, could affect the production of antibodies during the secondary immune response. For these studies, mice were immunized (day 0) and boosted (day 21) prior to the in vitro antigen recall assay, where cells were restimulated for 24 h in the presence of the T-specific antigen DNP-KLH. We compared levels of marginal-zone B cells (CD19+/IgM^high^/IgD^low^) in the ERK5*^f/f^* and ERK5*^f/f^* CD4-Cre mouse spleens, bone marrows, and lymph nodes (Figure 6B), as well as levels of activated B cells (B220+CD69+CXCR4-) in the bone marrow (Figure 6C). We found that independent of the site, the levels of marginal-zone B cells and activated B cells were equivalent between ERK5*^f/f^* and ERK5*^f/f^*CD4-Cre mice (Figure 6), even after re-stimulation in vitro. In addition, we examined levels of follicular helper T cells (T_fh_) (CD4+PD-1+CXCR5+) within the lymph nodes and found no significant difference in the percent of these cells independent of expression of ERK5. From these results, we inferred that ERK5 expressed in T cells did not lead to significant increases in activated or memory B-cell populations or in T_fh_ cell populations.

### 2.6. ERK5 Promotes Chemotactic Migration via CCL19

**Figure 7:**
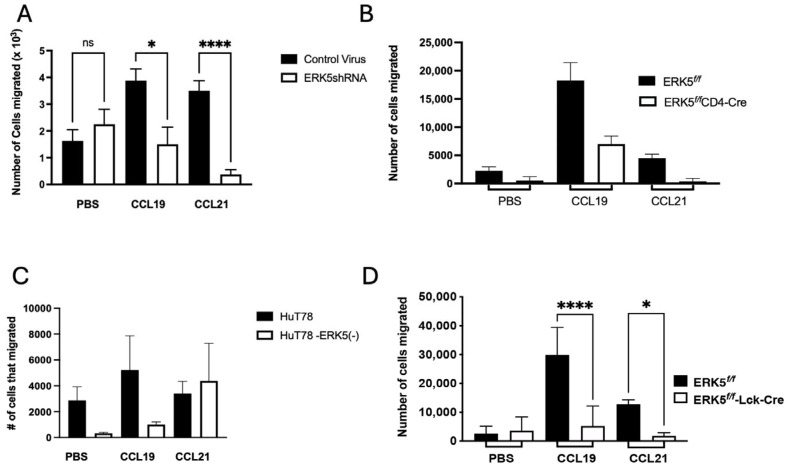
ERK5 promotes migration of T cells to CCR7 ligands. (**A**) Primary murine CD4+ cells were transduced with ERK5shRNA or control shRNA, and 96 h later, cells were induced to migrate to PBS (control), 40 nM CCL19, or 40 nM CCL21. Each column is the X ± SD of the number of cells that migrated across eight transwells over 90 min. (**B**,**D**) Primary CD4+ splenocytes were isolated from the spleens of ERK5*^f/f^* or ERK5*^f/f^*CD4-Cre mice or ERK5*^f/f^* or ERK5*^f/f^*Lck-Cre mice and induced to migrate to 40 nM CCL19 or CCL21 over four hours. (**C**) HuT78 human T cells were stably transduced with ERK5-targeted CRISPR-Cas9, selected in puromycin, and allowed to migrate to 40 nM CCL19 or CCL21 over four hours. Each column is the X ± SD migration of cells from *n* ≤ 3 independent experiments, isolated from two mice. Paired Student’s *t*-tests of ≥3 independent studies were performed to determine significance of differences between ERK5 (-) cells and controls (* *p* < 0.05, **** *p* < 0.0001, ns = difference between compared groups was not significant).

Pertussis toxin, which catalyzes ADP-ribosylation of Gα_i_ subunits, has been shown to enhance the expression of IgG1 during immunization [33]. Notably, pertussis toxin inhibits chemokine-induced chemotaxis, and along with other labs, we have used it to generate autoimmune disease in animal models [20,34,35,36]. In the absence of CCR7, T cells fail to migrate to CCR7 ligands, CCL19 and CCL21 [37]. We have shown that downstream from CCL19/CCR7-induced upregulation of ERK5, KLF-2 is upregulated [8]; KLF-2 is a transcription factor that promotes both thymocyte and T-cell migration [38]. ERK5 can both positively and negatively control migration of cells [39,40,41]; therefore, we questioned whether ERK5 promotes migration of T cells via CCR7/CCL19 to limit the production of IgG1. For these studies, we used two approaches: first, comparing the migration of primary murine CD4 cells transduced with ERK5shRNA, and second, assessing migration of T cells from ERK5*^f/f^* mice and ERK5*^f/f^*-CD4Cre mice or ERK5*^f/f^* or ERK5*^f/f^* Lck-Cre mice (Figure 7). CRISPR-Cas9 deletion of ERK5 in human HuT78 T cells only inhibited migration to CCL19 but not CCL21 (Figure 7C). In both cases, transwell migration assays showed that reduction in ERK5 and complete loss of ERK5 significantly reduced T-cell migration to either CCL19 or CCL21, indicating that ERK5 activation is necessary for CCR7-induced T-cell chemotactic migration.

## 3. Discussion

The major finding in our study is that we have identified a novel role for CCL19 in T-cell attenuation of the levels of IgG1 generated during the humoral adaptive immune response. Loss of the CCR7 ligand, CCL19, and the resultant loss of downstream signaling through ERK5 resulted in enhanced production of IgG1 and, at times, IgG2c as well, demonstrating a role for a signal cascade between CCR7, CCL19, and ERK5 that limits levels of IgG1 generated during the adaptive humoral immune response. In previous studies, we have shown that following binding to CCL19, signaling through CCR7 via ERK5 leads to upregulation of S_1_P_1_ expression and controls keratinocyte cell proliferation [15]. Since we found similar numbers of B or T lymphocytes in the lymph nodes of ERK5*^f/f^*CD4-Cre mice compared to ERK5*^f/f^* mice, and were unable to find significant differences in proliferation, we inferred that any contribution that ERK5 may have in regulating cell proliferation does not play a significant role in limiting the levels of IgG1 [17]. Notably, ERK5 is not required for T-cell development; T-cell numbers in the spleen and lymph nodes of ERK5*^f/f^*CD4-Cre mice are the same as those of their wild-type littermates. In addition, mRNA levels of CCR7 are unaffected in ERK5*^f/f^*CD4-Cre mice compared to wild-type littermates [42].

In CCR7^−/−^ mice, we found increases in IgG1, IgG2b, IgG2c, and IgG3 levels when compared to wild-type control mice, possibly due to the persistence of antigen-specific T cells within priming sites, which is distinct from the isotypes reported for the BALB/c mice [4,43]. Since the roles of ERK5 in inflammation, which signals downstream from CCR7/CCL19, have been controversial, we examined the role of ERK5 in limiting production of IgG1 (Figure 4). We observed that while signaling through ERK5 limits levels of IgG1, we did not see a significant change in the levels of IgG2c or IgG2b, suggesting that alternative signaling pathways are activated to induce isotype switching within these classes that may be downstream of CCR7 activation by its other ligand, CCL21.

Antibody immune responses can be thymus-dependent (TD) or thymus-independent (TI). Although the T-cell adhesion to antigen-presenting cells, which normally upregulate CCL19, is limited, we expect that T cells still form conjugates with dendritic cells, albeit to a significantly lesser extent (Figure 3). TI type 2 antigens, which are repetitive and capable of activating B cells without T help, cross-link B-cell receptors and lead to production of mostly low-affinity IgM; we did not see any effect on the levels of IgM. The B cells found within the peripheral lymph nodes of CCR7^−/−^ and paucity of lymph node T cells (plt) mice that lack CCL19 and CCL21ser have an activated phenotype and display increased surface expression of the activation markers CD86 or MHC-class II and the low-affinity Fcε (CD23) [43]. Moreover, CCR7^−/−^ B cells prime CD4+ T cells more efficiently than wild-type cells, likely due to more efficient antigen processing relative to wild-type cells [43].

When we examined the activation state of lymphocytes during an in vitro rechallenge assay (Figure 6A), following immunization and boosting of mice in vivo, we found no difference between the levels of activated T or B lymphocytes independent of ERK5 expression (Figure 6). Although IL-4 is an important cytokine in the regulation of the B-cell immune response, and IL-2 in regulating the T-cell immune response, we did not find a significant difference in the levels of any of the cytokines that were present following a rechallenge assay (Figure 5). We expect that the B cells produced elevated levels of IgG1 and IgG2c, since these isotypes remained elevated independent of the method used to disrupt CCL19 signaling. Since CCL19 is upregulated on activated dendritic cells, this could indicate that in the absence of dendritic cell CCL19 or its downstream effector ERK5, T cells are stimulated by other antigen-presenting cells such as B cells within lymph nodes that recruit T cells following T-cell upregulation of CXCR5 and the expression of CXCL13 within the B-cell follicles.

Disruption of leukocyte migration downstream using pertussis toxin has been linked to increased levels of IgG1 and, like our results, has no effect on IgG2a/c, IgG2b, IgG3, or IgM [33]. Pertussis toxin disrupts chemotactic migration to CCL19 [8], and chemotaxis via CCR7 was not present in T cells lacking CCR7, its ligand CCL19, or ERK5. In addition, anti-IL-4 had no effect on the IgG1 levels during the primary immune response downstream from pertussis toxin [33] even though Th2-cell production of IL-4 can enhance secretion of IgG1. These observations are in line with our studies, where we see no significant differences in the levels of IL-4 between control and ERK5-deficient mice.

In summary, our results suggest that loss of the CCL19/CCR7 signaling axis that inhibits CCL19 chemotaxis induces high levels of IgG1 during the secondary adaptive immune response, independent of increased levels of IL-4, but instead due to the presence of T cells that are unable to migrate to CCL19. Our data do not exclude additional roles for ERK5, which could regulate its long-term effects on survival or changes in the T-cell effector state, particularly since ERK5 is activated by many diverse ligands. Moreover, since we have found that exposure to a CCL19 antagonist can produce an enhanced immune response, future studies will focus on defining the activation state of other CCR7-expressing APCs in the immune system and the cytokines that may contribute to this enhanced immune response.

## 4. Materials and Methods

### 4.1. Mice and Reagents

C57BL/6 mice (#000664) were purchased from Jackson Labs, Bar Harbor, ME, USA. CCL19_8-83_ was custom-synthesized and purchased from United Biosystems Inc. (Rockville, MD, USA) or from Pepmic (Suzhou, China). ERK5*^f/f^* mice were a generous gift from Dr. Cathy Tournier (University of Manchester, Manchester, UK) [16], and CCR7*^−/−^* (Jackson Labs#006221), Lck-Cre (Jax mice #003802), and CD4-Cre (Jax mice #022071) mice were purchased from the Jackson Laboratory and acclimatized at least 7 days before use. All experiments were approved by IACUC at The University of Texas at El Paso (A201310) and at The University of Kansas Medical Center (2010-1867).

### 4.2. Induction of a Humoral Immune Response in Mice

Age- and sex-matched 6–8-week-old (C57BL/6 (CCR7^+/+^) (control)), vs. C57BL/6 CCR7^−/−^ or C57BL/6 ERK5*^f/f^* (control) vs. C57BL/6 ERK5*^f/f^*CD4-Cre, C57BL/6-CD4-Cre (control), C57BL/6 ERK5*^f/f^*Lck-Cre or Lck-Cre (control), or ERK5*^f/f^* (control) mice were used for these studies. Both males and females were used and randomized by mixing cages; all cages were located in the same area of the vivarium. No other criteria were set. Where indicated, wild-type mice were pre-treated with 200 µg of CCL19_8-83_ or anti-mCCL19 (R&D; MAB880, Minneapolis, MN, USA) i.p. [30] 24 h prior to antigen injection. Mice were treated again with 200 µg of CCL19_8-83_ at boost. The IC_50_ for the CCL19 antagonist is 692 nM [30]. All mice were immunized by injection of 200 µg of DNP-KLH (Calbiochem, Burlington, MA, USA)/200 µL of ddH_2_O (dissolved overnight with rotation) at the base of the tail (100 µL/side of the base) and then boosted with the same technique with 200 µg of DNP-KLH three weeks later. On dates indicated, blood was isolated from submandibular bleeds and collected into a heparinized tube using a fresh, sterile 4 mm Lancet for each animal. Serum was collected following centrifugation, and isotypes of DNP-specific antibodies present in the sera were determined by ELISA. Following the completion of the study, the mice were euthanized with Euthasol (Virbacah, West Lake, TX, USA) or Beuthanasia, (Merck Animal Health, Rahway, NJ, USA). Once mice were insensitive to toe pinch, the mice were cervically dislocated, and the chest cavities were opened prior to tissue collection. Between 4 and 7 mice were used per group, and the numbers of mice used in the study were based on a Power Analysis of data generated from pilot studies and published work [4].

### 4.3. ELISA Measurements of Antibody Levels

NUNC MaxiSorp ELISA plates (Thermo Fisher Scientific, Waltham, MA, USA; #44-2404) were coated overnight with DNP-BSA (Calbiochem, San Diego, CA, USA; #44-2404) dissolved in a sterile filtered 200 mM sodium carbonate/bicarbonate pH 9.4 buffer. Plates were blocked with sterile filtered 50 mM carbonate/bicarbonate buffer and coated with 50 mM carbonate/bicarbonate buffer 20 µg/mL DNP-BSA overnight at 4 °C, rinsed with PBS–surfactant (diluted from 10 × PBS BP3994; Thermo Fisher Scientific/0.5% Tween-20 (P9416; Sigma-Aldrich, St. Louis, MO, USA)), and blocked with PBS/2%BSA (Gold Biotechnology, Olivette, MO, USA, #A420100). For serum measurements of IgG1, sera were diluted at 1:10,000 or 1:5000; to measure levels of IgG2b, IgG2c sera were diluted at 1:1000, and to measure levels of IgG3, serum dilutions of 1:1000, 1:100, or 1:10 were used. Anti-DNP antibody, clone 9H8.1 (MAB2223, EMD Millipore, Burlington, MA, USA), was used as the standard for serial dilution into PBS/1%BSA. Dilutions of immunized mouse serum or control anti-DNP antibody standards were incubated on DNP-BSA-coated plates at room temperature for 1 h. Plates were washed, loaded with rabbit anti-mouse (IgG1 (SA510213; Invitrogen, Thermo Fisher Scientific, Waltham, MA, USA), IgG2b (SA510210; Invitrogen), IgG2c (SA510221; Invitrogen), or IgG3 (SA510220; Invitrogen)) antibody at a dilution of 1:5000, and incubated at room temperature for 1 h. Goat anti-rabbit Ig-G-HRP (#31460, Invitrogen, Carlsbad, CA, USA) was added at 1:4000, in PBS–surfactant, rinsed in 1×PBS, and incubated with 1-Step^TM^ TMB ELISA Substrate (#34028 Thermo Fisher Scientific, Waltham, MA, USA ) for 10 min at room temperature. Plates were read at 655 nm, and the reaction was stopped with 2 M sulfuric acid, and absorbance was measured at 450 nm.

### 4.4. Receptor Internalization Assay

HuT78 (T1B-161; ATCC, Manassas, VA, USA) cells were collected by centrifugation (5 min at 90× *g* at RT); the supernatant was decanted, resuspended in 0.5 mL of serum-free RPMI (*sf* RPMI), and incubated at 37 °C for 10 min prior to assay. One hundred microliters of cells was transferred to a second tube, labeled “0 min”, and placed back in the water bath. Ligand was added to the 400 µL of cells remaining in the first tube at a concentration of 200 nM. At the indicated time points, cells were transferred from the first tube to 1 mL of ice-cold *sf* media and allowed to rest on ice. The final time point and the “0” time point were collected at the same time, and the cells were allowed to rest on ice for 30 min. Cells were pelleted (5 min at 90× *g* at 4 °C), and supernatants were decanted. Cells were washed with ice-cold *sf* media 3 times, ending with the cells in a pellet. The cells were resuspended in 20 µL of staining buffer (1 µL of anti-human CCR7 (#150503R; R&D/Thermo Fisher Scientific)/1 µL of CD16/32/18 µL of PBS/2%BSA) for 20 min on ice, with volumes adjusted to 200 µL with ice-cold staining buffer without antibody and analyzed by flow cytometry.

### 4.5. T-Cell Isolation

Murine splenocytes were harvested from experimental mice by dissociation of isolated spleens by pressing the tissue through a metal mesh with the rubber end of a syringe, red blood cells were lysed with ACK lysis buffer (Lonza, Walkersville, MD, USA), and T cells were purified by negative selection (EasySep, Stemcell Technologies, Vancouver, BC, Canada). Isolated T cells were either used immediately for assays or maintained in T-cell media [RPMI-1640 (Thermo Fisher Scientific, Waltham, MA, USA), 10% heat-inactivated fetal bovine serum (Hyclone, Wilmington, DE, USA), 2 nM L-glutamine (Invitrogen, Thermo Fisher Scientific, Waltham, MA, USA), 50µM β-mercaptoethanol (Thermo Fisher Scientific, Waltham, MA, USA), 20 U/mL IL-2 (A generous gift from Dr. Christophe Nicot, University of Kansas Medical Center-Department of Pathology), 10 U/mL IL-7 (R&D Systems, Thermo Fisher Scientific, Waltham, MA, USA), 100 U/mL penicillin/100 µg/mL streptomycin (Cellgro Scientific, Los Angeles, CA, USA) in a humidified atmosphere at 37 °C and 5% CO_2_ for use within 3 days of isolation.

### 4.6. Generation of Bone-Marrow-Derived Dendritic Cells

Bone-marrow-derived dendritic cells (BMDDCs) were generated from C57BL/6 wild-type mice as described [8]. Briefly, femurs were isolated from C57BL/6 wild-type mice, and bone marrow was flushed with RPMI using a 27 or 30 G needle attached to a syringe. Cells were diluted ten-fold into Trypan Blue (Sigma-Aldrich, St. Louis, MO, USA)/5% acetic acid and counted on a hemacytometer. Then, 2 × 10^6^ bone marrow cells were plated on 100 mm bacterial Petri dishes and cultured in 10 mL of DC media [RPMI 1640 (Cellgro Scientific, Los Angeles, CA, USA), 10% heat-inactivated fetal bovine serum (Atlanta Biologicals, R&D Systems), 100 µg/mL penicillin–streptomycin (Cellgro Scientific, Los Angeles, CA, USA), 50 µM β-mercaptoethanol (Thermo Fisher Scientific, Waltham, MA, USA), 20 ng/mL murine granulocyte macrophage colony-stimulating factor (recombinant murine GM-CSF) (Shenandoah Biotechnology, Warwick, PA), and 2 nM L-glutamine (Cellgro Scientific, Los Angeles, CA, USA)]. After 48 h, 10 mL of fresh DC media was added to each dish. Two and six days later, 10 mL of supernatant/cells was removed, cells were collected by centrifugation (90× *g*) for 5 min at room temperature, and the pellet was resuspended with fresh, pre-warmed DC medium. On day 9, non-adherent DCs were collected and used immediately. Alternatively, DCs were frozen over 4 h at −80 °C in 10% DMSO/10% heat-inactivated fetal bovine serum/80% DC media and stored in a liquid nitrogen vapor freezer until use.

### 4.7. Chemotaxis Assays

Human T cells (CEM) were grown in 10% heat-inactivated fetal bovine serum/RPMI/2 mM L-glutamine (cRPMI) in a humidified 5% CO_2_/air environment, isolated from culture, and allowed to migrate for 90 min across a 5 µm^2^ pore membrane, pre-coated with 10 µg/mL human fibronectin (Sigma-Aldrich, St. Louis, MO, USA). Then, 2 × 10^5^ cells were loaded in the top well of a neuroprobe chemotaxis chamber (NeuroprobeGaithesburg, MD, USA) in the presence of cRPMI and induced to migrate to 10 nM CCL19/RPMI in the presence of CCL19_8-83_ at the indicated concentrations and allowed to migrate for 90 min. The membranes were stained, and the number of cells that migrated was assayed. Data is presented as mean ± SE and is pooled from three independent experiments.

### 4.8. DC/T-Cell Adhesion Assay

DCs were labeled with (10 µM) 5-(and-6)-(((4-chloromethyl)benzoyl)amino) tetramethyl-rhodamine ((CMTMR) Thermo Fisher Scientific, Waltham, MA, USA) for 20 min at 37 °C, washed 3× in PBS/2%FBS, primed with 50 µg/mL ovalbumin, and matured overnight with 100 ng/mL TNFα. If frozen DCs were used, to initiate cultures, cells were thawed at 37 °C, collected by centrifugation (5 min at 90× *g*), and allowed to recover overnight in DC media at 37 °C in a humidified 5% CO_2_ incubator. After 16–24 h of recovery, cells were matured in the presence of 100 ng/mL TNFα and primed with 50 µg/mL ovalbumin ± 0.1 µg/mL CCL19_8-83_ for 24 h. Peripheral blood was used as the source of C57BL/6-Tg(OT-II) (Taconic Biosciences, Germantown, NY, USA) leukocytes. To this end, blood was collected (100 µL) by mandibular bleed into heparinized tubes; red blood cells were lysed in ACK lysis buffer (Thermo Fisher Scientific, Waltham, MA, USA) at 37 °C for seven minutes and then adjusted with 3 volumes of complete media. C57BL/6-Tg (OT-II) T cells were collected by centrifugation (5 min at 90× *g*), and the resultant pellet was labeled with (5 µM) 5-(and-6)-carboxyfluorescein diacetate, succinimidyl ester (CFSE, Thermo Fisher Scientific, Waltham, MA, USA) for 10 min at 37 °C in a water bath. The cells were washed 3 times with PBS/2%FBS and plated with ovalbumin-primed, CFSE-labeled mature dendritic cells in DC media. The concentration of CCL19_8-83_ in the DCs was brought to 1.1 µM by the addition of 5 µL of 1.1 mM CCL19_8-83_ into 5 mL of media. The vehicle control was 5 µL of PBS in 5 mL of media. Cells were allowed to form conjugates for 24 h and fixed by adjusting the media to 2% paraformaldehyde (Thermo Fisher Scientific, Waltham, MA, USA) and allowing the conjugates to fix for a minimum of 30 min. Unbound cells were removed by rinsing, random fields containing dendritic cells were imaged, and the number of T cells bound to DCs was counted per 100 dendritic cells.

### 4.9. Recall Assays

Mice were immunized by injection of 200 µg of DNP-KLH (Calbiochem, San Diego, CA, USA)/200 µL of ddH_2_O (dissolved overnight with rotation) at the base of the tail (100 µL/side of the base) and then boosted with the same technique with 200 µg of DNP-KLH three weeks later. Nine days later, mice were euthanized, spleens removed, and splenic mononuclear cells were disaggregated through metal meshes; single cells were collected by passing through screens, counted, and seeded at a density of 2 × 10^6^ cells per well for spleen in 10% heat-inactivated fetal bovine serum/RPMI. Cells were challenged with 10 µg/mL DNP-KLH overnight (for 16–24 h). Cells were collected, stained, and analyzed by flow cytometry, or media was collected and assayed using a Cytomix^TM^ bead assay (Milltenyi, Gaithesburg MD, USA) per manufacturer’s directions.

### 4.10. Fluorescence-Activated Cell Sorting (FACS) Analysis of Lymphocytes

Expression of markers in T cells was measured using a Gallios Flow Cytometer Analyzer (Beckman Coulter Diagnostics, Brea, California, USA) following in vitro rechallenge. Expression of proteins was visualized using fluorescently labeled antibodies. Single cells were stained in staining buffer (RPMI/10% Normal rat serum (Jackson Labs, Bar Harbor, ME, USA 012-000-120), 2% CD16/32 (BD-B553142)/2% Normal Mouse serum (Jackson Labs, Bar Harbor, ME, USA 015-000-120)) with fluorophore-conjugated antibodies on ice for 20 min or for the anti-CCR7 stain at 37 °C. Cells were pelleted and rinsed 3× in PBS/1% BSA. Live cells were gated for side scatter and forward scatter. Single-stained cells or fluorescence minus one (FMO) cells were used as controls.

### 4.11. Statistical Analysis

Results were calculated as means ± S.E. *p* values of < 0.05 were considered statistically significant, as calculated by an unpaired, two-tailed *t*-test (Prism version 10.6.0).

## Data Availability

All data for this study is archived at the University of Texas at El Paso and is available from CMV upon request.

## Abbreviations

The following abbreviations are used in this manuscript:

CCL19	C-C chemokine ligand 19
CCL19_8-83_	Amino acids 8-83 of murine CCL19; a CCL19 antagonist
CCL21	C-C chemokine ligand 21
CCR7	C-C chemokine receptor 7
Cre	Cre recombinase
DNP-KLH	Dinitrophenyl-keyhole limpet hemocyanin
EBI	Epstein-Barr virus-induced molecule 1
EDG-1	Endothelial differentiation gene 1
ERK5	Extracellular signal-regulated kinase 5
Flox	Flanking *loxp* sites
Lck	A T-cell-specific src family kinase that is expressed in the thymus
